# Measuring the availability and geographical accessibility of maternal health services across sub-Saharan Africa

**DOI:** 10.1186/s12916-020-01707-6

**Published:** 2020-09-08

**Authors:** A. S. Wigley, N. Tejedor-Garavito, V. Alegana, A. Carioli, C. W. Ruktanonchai, C. Pezzulo, Z. Matthews, A. J. Tatem, K. Nilsen

**Affiliations:** 1 WorldPop, Geography and Environmental Science, University of Southampton, Highfield Campus, Southampton, SO17 1BJ UK; 2 Population Health Unit, Kenya Medical Research Institute - Wellcome Trust Research Programme, P.O. Box 43640, Nairobi, 00100 Kenya; 3 Faculty of Science and Technology, Lancaster University, Lancaster, LA1 4YR UK; 4 Division of Social Statistics and Demography & Centre for Global Health, Population, Poverty and Policy, Faculty of Social and Human Sciences, University of Southampton, Southampton, SO17 1BJ UK

**Keywords:** Emergency obstetric care, Universal health coverage, Healthcare accessibility, Maternal and newborn health, GIS

## Abstract

**Background:**

With universal health coverage a key component of the 2030 Sustainable Development Goals, targeted monitoring is crucial for reducing inequalities in the provision of services. However, monitoring largely occurs at the national level, masking sub-national variation. Here, we estimate indicators for measuring the availability and geographical accessibility of services, at national and sub-national levels across sub-Saharan Africa, to show how data at varying spatial scales and input data can considerably impact monitoring outcomes.

**Methods:**

Availability was estimated using the World Health Organization guidelines for monitoring emergency obstetric care, defined as the number of hospitals per 500,000 population. Geographical accessibility was estimated using the Lancet Commission on Global Surgery, defined as the proportion of pregnancies within 2 h of the nearest hospital. These were calculated using geo-located hospital data for sub-Saharan Africa, with their associated travel times, along with small area estimates of population and pregnancies. The results of the availability analysis were then compared to the results of the accessibility analysis, to highlight differences between the availability and geographical accessibility of services.

**Results:**

Despite most countries meeting the targets at the national level, we identified substantial sub-national variation, with 58% of the countries having at least one administrative unit not meeting the availability target at province level and 95% at district level. Similarly, 56% of the countries were found to have at least one province not meeting the accessibility target, increasing to 74% at the district level. When comparing both availability and accessibility within countries, most countries were found to meet both targets; however sub-nationally, many countries fail to meet one or the other.

**Conclusion:**

While many of the countries met the targets at the national level, we found large within-country variation. Monitoring under the current guidelines, using national averages, can mask these areas of need, with potential consequences for vulnerable women and children. It is imperative therefore that indicators for monitoring the availability and geographical accessibility of health care reflect this need, if targets for universal health coverage are to be met by 2030.

## Background

The last few decades have seen substantial global reductions in maternal and neonatal mortality [1], with the maternal mortality ratio (MMR) falling by almost half since the establishment of the Millennium Development Goals [2]. However, despite progress, many countries fell far short of targets, with some seeing little or no change [3]. Of an estimated 295,000 maternal deaths occurring globally in 2017, approximately 66% (196,000) of these were in sub-Saharan Africa (SSA) alone, and it is here, where the maternal mortality rate is the highest, that data on births and deaths is often the least robust [4]. Moreover, inequalities in maternal and neonatal health outcomes are not just limited to between countries, but exist within countries as well, presenting fundamental barriers to progress, particularly among the most disadvantaged population groups [1, 5, 6].

Reducing mortality relies on the provision of high-quality care, and a key emphasis of the 2030 Sustainable Development Goals (SDG) is the achievement of universal health coverage (UHC) [7]. Monitoring progress is key to achieving targets and the World Health Organization (WHO) provide guidelines for estimating the availability and use of emergency obstetric care (EmOC) services [8]. One limitation of these indicators is that they do not consider the geographical accessibility of such services, regarding the time it takes to get to a facility, where the availability of a service may be limited by the number of women who can reach and use it [9]. This limitation is a key contributing factor in positive maternal health outcomes, with the delay in reaching care one of the key determinants in a women’s ability to receive the services they need [10]. However, awareness is growing on the need to improve geographical access to emergency care, and a Lancet commission on global surgery defines geographical accessibility as the proportion of the population that can access, within 2 h, a facility with essential surgical and anaesthesia services, with a target of 80% minimum coverage by 2030 [11].

With an increasing focus on the coverage and accessibility of health services and access to data at increasing spatial resolutions, the use of GIS in maternal health research is growing [12], and several studies identify the need to evaluate the EmOC guidelines [13–15], seen as ‘too general and inconsistent’ [13]. Douangphachanh et al. [16] consider the use of population density, while Bosomprah et al. [17] suggest the use of births and pregnancies would be a more accurate representation of the needs of a population. This is further explored in Ebener and Stenberg [18] where births are used in place of population to assess geographic accessibility to services, and more recently, in Ebener et al. [19] who propose standardised geographical indicators of physical access to emergency obstetric and newborn care for low-income and middle-income countries [19].

Further to this, ancillary data, such as travel-time, elevation, roads, and rivers, are considered important when considering the geographical distribution of services [12, 18, 20], with a variety of spatial modelling techniques employed across a number of studies [21–23]. Methods range from the more simple modelling of Euclidean distances [21] and road network analysis [24], towards a trend of more sophisticated models of accessibility, considering, additionally, the effects of topography, land-cover, and means of transportation in the estimation of travel time to the nearest facility [18, 20]. Ouma et al. [25] model the accessibility of hospitals for women of childbearing age (WoCBA), using a gridded travel-time surface, where each cell represents the cumulative time required to cross pixels in the least cost path. In this, travel-time is modelled across SSA, with the development of the first geocoded inventory of public hospitals with emergency services [26].

Despite guidelines to assess the sub-national coverage of services [27], estimates are often calculated at the national level, which can be misleading [13], and research is limited in the measurement of these indicators at sub-national levels, where, at most, only a handful of countries are considered [18, 28]. This is likely due to a previous lack of spatial data at these smaller administrative levels. Additionally, they do not consider the accessibility of services, regarding the time taken to reach a hospital or the actual population at risk, for example, WoCBA, pregnancies, or births [17–19]. Using high-resolution estimates of population and pregnancies [29–31], hospital locations, and their associated travel times [32], we estimate the availability and geographical accessibility of services across SSA and assess the suitability of these indicators for monitoring maternal health targets. In the context of these estimates, we evaluate how the guidelines for monitoring the availability and use of emergency obstetric care could be revised, to more accurately reflect the target population, accessibility of services, and equitable access to care. As stated in the guidelines [27], we use population to estimate the availability of services, and pregnancies for measuring physical access to emergency obstetric and newborn care in low-income and middle-income countries, as proposed by Ebner et al. [19]. This is carried out at both national and sub-national levels to explore how different classifications of coverage [9], varying spatial scales and input data can significantly impact monitoring outcomes.

## Methods

### Data

**Fig. 1:**
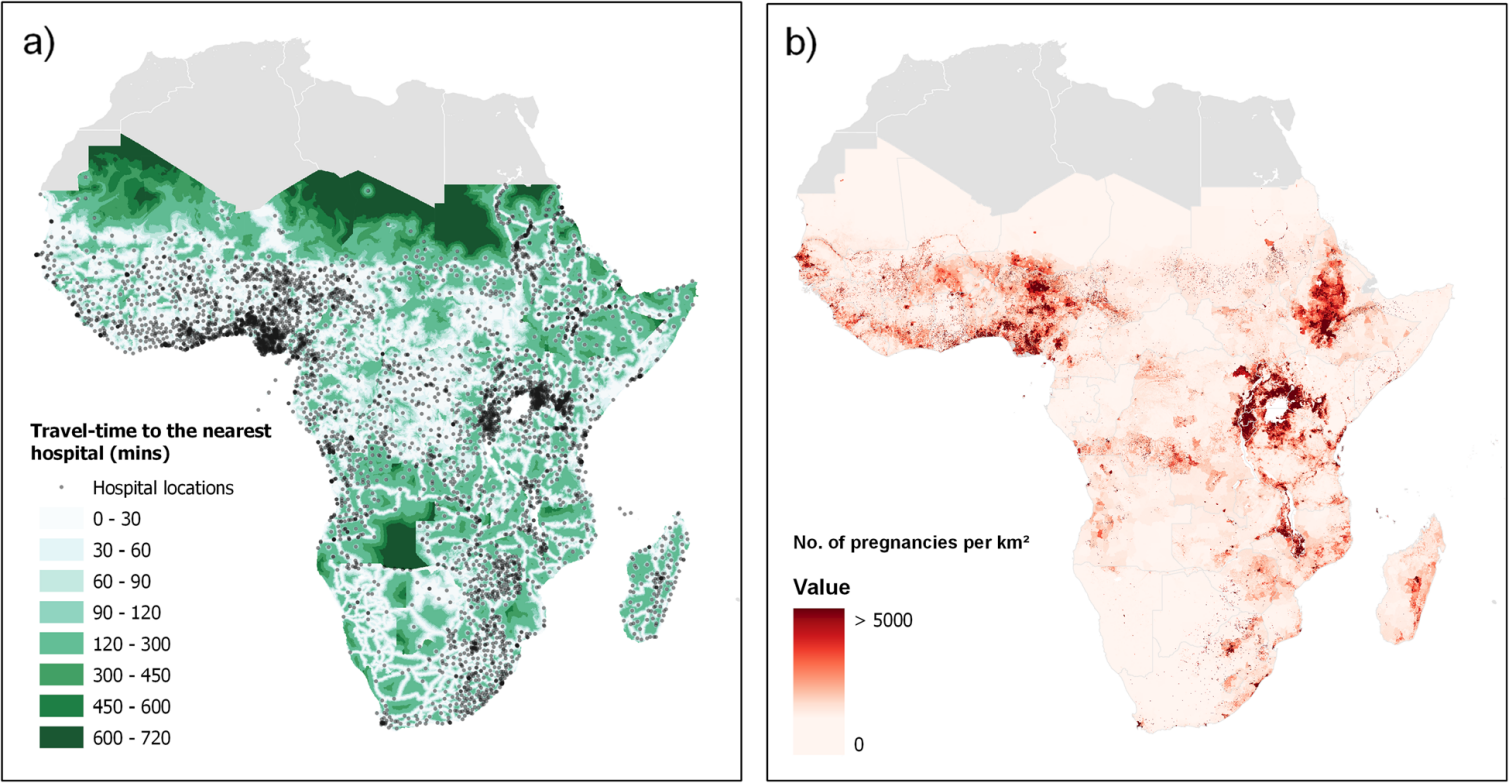
Map **a** showing travel-time to the nearest hospital and map **b** showing the estimated number of pregnancies in 2015 per km^2^

Hospital location data, described in a recent study of geographical accessibility to ‘emergency hospital care provided by the public sector’ across SSA [20], was obtained from the Harvard Dataverse [26]. Compiled from various sources, including ministries of health, health management information systems, and government statistical agencies, the data represents hospital services targeted at a broad range of emergency or referral care to the general population [20]. The data includes all hospitals managed by national and local governments, faith-based, and non-governmental organisations, where the facility is the main provider of emergency care and governed by national health guidelines and regulations. Private hospitals and hospitals providing only specialised services are not included in the data, considering the availability of and accessibility to public services only, though this may cause limitations in countries such as the Democratic Republic of the Congo where many rely on access to private health care [33]. Additionally, many hospitals throughout SSA do not provide all signal function functions required to be classified as a CEmOC facility, and information on their CEmOC status is not readily available [29]. It is therefore assumed that hospitals provide the signal functions defined in the guidelines for the provision of comprehensive emergency obstetric care (CEmOC). This data set forms the basis of the analysis and includes forty-eight countries across SSA (Fig. 1a).

Gridded estimates of population and pregnancies were obtained at 1 km spatial resolution from the WorldPop database for 2015 [34] (Fig. 1b shows the estimated number of pregnancies per km^2^ across SSA). Detailed methods are described elsewhere [29–31], though to summarise briefly, population estimates are first derived using the most recently available data at the lowest available administration unit, using a semi-automated dasymetric modelling approach, then disaggregated further by age and sex, to determine numbers of WoCBA. Country respective fertility rates are then applied, using the most recently available data on stillbirths, miscarriages, and abortions, to estimate the numbers of pregnancies per 1 km grid cell. It is important to note, that while the population estimates can be determined to a relatively good degree of accuracy, information on sub-national birth rates are much less widely available, and sub-national estimates of pregnancies, abortions, and stillbirths do not currently exist. The method therefore relies on the input of national estimates to determine these rates and an assumption of no within-country variation [35].

Geographical accessibility was estimated by considering the travel time to the nearest hospital, using a gridded travel-time surface [32], where each cell represents the cumulative time required to cross pixels in the least cost path. This surface was generated using AccessMod5 [36], a WHO tool used for modelling geographical accessibility to health care. Detailed methods are described elsewhere [37], though, in summary, the surface is created by modelling the effects of topography, available transport networks, land-cover and barriers to travel, using cost-distance analysis, with a composite of walking and motorised travel speeds and the set of geo-located hospitals [26]. This model assumes motorised travel along the road network, assigning 80 km/h on primary roads, 60 km/h on secondary roads, and 10 km/h on tertiary roads, with all other non-road cells assigned a speed of up to 5 km/h, depending on the land-cover type and slope, and assuming patients could walk, were carried, or transported by other means [38]. However, where access to motorised travel is not available, and where seasonal variations can impact road conditions, this model may largely overestimate the time taken to reach the nearest hospital [39, 40]. The model is also confined to national borders, assuming patients do not cross the border to reach the nearest hospital, though not constrained by sub-national zoning. Figure 1b) shows both the gridded travel-time data and hospital locations.

The Global ADMinistrative areas (GADM) database [41] was used to define administrative units at both national and sub-national levels. As terminology varies across countries, different administrative levels are referred to using standardised abbreviations. National boundaries are referred to using the country name or ISO code [42] and sub-national boundaries using adm-1 or adm-2, where adm-1 typically represents the state or province level and adm-2 the district or county level. Where sub-national boundaries were not available, the country was not analysed at these levels. The gridded travel-time surface was used to select the countries to use for the analysis, with 45 countries analysed at the national and adm-1 levels and 43 at adm-2, where adm-2 level boundaries for Cape Verde and Lesotho were not available.

### Analysis

The availability of services was defined as the number of hospitals per 500,000 population, with a minimum target of one comprehensive facility for every 500,000 population. This was carried out at both national and sub-national levels, assuming hospitals provide the comprehensive care set out in the guidelines [8]. Estimates were calculated by multiplying the number of hospitals within a defined administrative unit, by 500,000, and zonal statistics used to determine the population and hospital counts for each unit. Where the number of hospitals per 500,000 population is greater than one, the minimum level of care is assumed to have been met. Following previous work [43], the percentage of the target achieved was subsequently calculated, to provide a measure of achievement at both national and sub-national levels. A combination of ArcGIS 10.4.2 [44], Python 2.7.10, and R version 3.5.0 [45] was used in the analysis.

Geographical accessibility was defined as at least 80% of pregnancies within an administrative unit located within 2 h to the nearest hospital [11, 19]. For this indicator, pregnancies were estimated instead of the general population [17, 19], to be more reflective of the population at risk. Using the gridded travel-time surface and the WorldPop pregnancies data sets, the indicator was calculated at both national and sub-national administration levels. Using the Arcpy Python package, the travel surface was reclassified into time zones, whereby all cells within a zone were classified by the value 1, and all others as 0, and each zone multiplied by the pregnancy data sets, to calculate the pregnancy counts for each of the time zones. Zonal statistics were used to calculate the numbers of pregnancies for each administrative unit, and outputs combined and reformatted using a combination of Python and R. From the resulting counts, their associated proportions were calculated, and the critical 2-h maximum travel time [8, 46] used to group the outputs into those ‘within 2-h travel time’ and those ‘greater than 2-h travel time’ from the nearest hospital, and the 80% minimum coverage target [11] used to define a boolean style classification. The outputs were joined to their associated administrative boundaries using ArcGIS.

With both indicators, it is important to note that their calculation relies on the use of defined administrative boundaries. This is limited in that it cannot account for the utilisation of cross border facilities and is highly sensitive in the partitioning of the administrative units. This is known as the Modifiable Areal Unit Problem (MAUP), which describes how spatial summary measures are inherently influenced by the administrative boundaries that they are reported at [47].

The results of the geographical accessibility analysis were then compared to those of the availability analysis to highlight those areas meeting the availability targets but not meeting the geographical accessibility targets, regarding the 2-h travel time [8, 46] and the 80% coverage threshold [11]. These were plotted and four classifications identified: (1) those not meeting the availability or geographical accessibility thresholds, (2) those meeting the availability threshold but not the geographical accessibility threshold, (3) those meeting the geographical accessibility threshold but not the availability threshold, and (4) those meeting both the availability and geographical accessibility thresholds. To facilitate comparability between countries of varying size and administrative units, the average spatial resolution (ASR) was calculated, as the square root of the country area (km^2^) divided by the total number of administration units, for each country at adm-1 and adm-2, classifying the countries into quartiles based on the minimum and maximum ASR values at both sub-national levels.

## Results

**Fig. 2:**
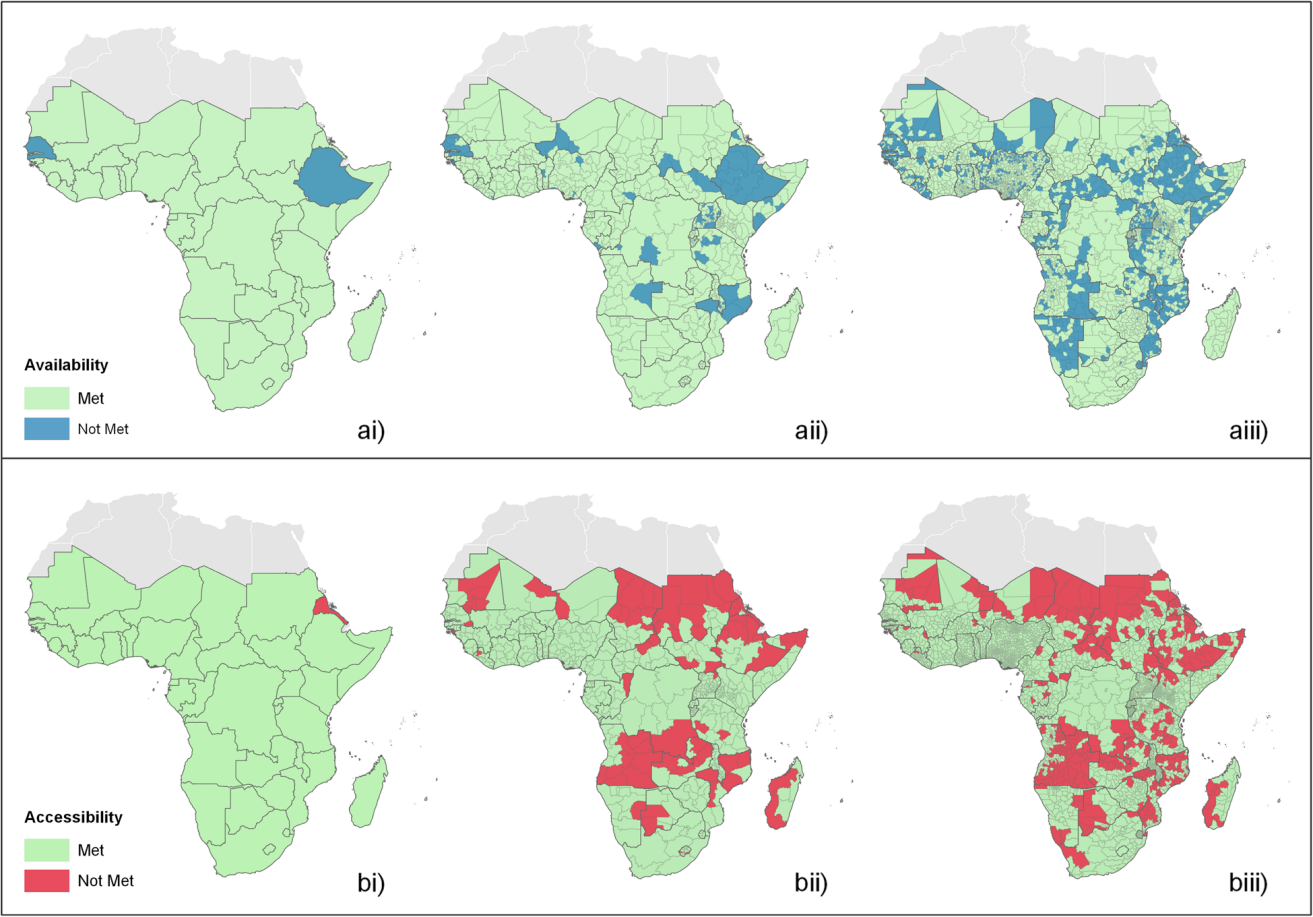
Maps showing where the availability target has not been met, at **ai** national, **aii** adm-1, and **aiii** adm-2 levels, and where the accessibility target has not been met at **bi** national, **bii** adm-1, and **biii** adm-2 levels

Indicators measuring the availability and geographical accessibility of hospitals were estimated at national and sub-national levels. The geographical distribution of these indicators is shown in Fig. 2, to highlight areas where targets have not been met.

**Fig. 3:**
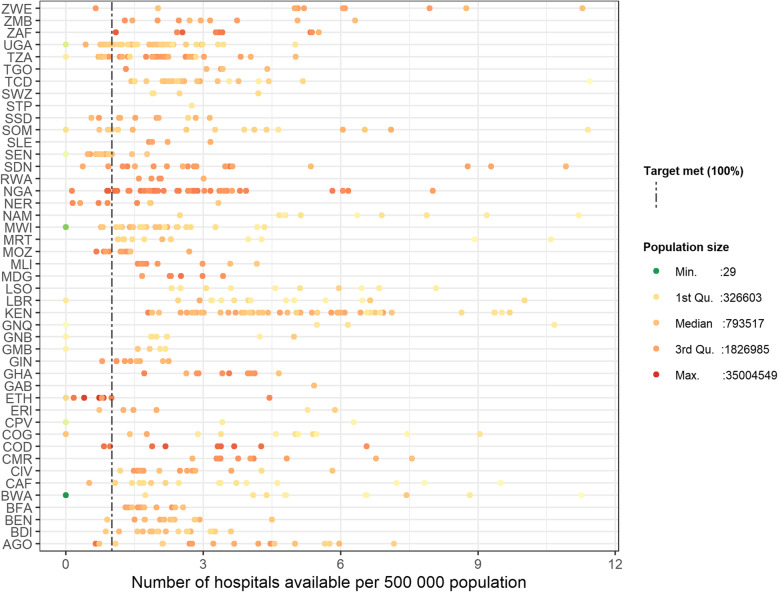
The number of hospitals available per 500,000 population, by country, for each adm-1 unit, classified by population size (with breaks at the minimum, 1st quartile, mean, 3rd quartile, and maximum values)

We estimated the availability of hospitals for 45 countries at the national and adm-1 administrative levels, and for 43 at adm-2 (Additional file 1). At the national level, all countries were found to meet the availability target (1 hospital per 500,000 population), with the exception of Ethiopia (77% of target) and Senegal (96% of target) (Fig. 2ai and Additional file 2). However, we identified substantial subnational variability, with 26/45 (58%) of the countries at adm-1 (Additional file 3) and 41/43 countries (95%) at adm-2 (Additional file 4) found to have at least one administrative unit not meeting this target. This variation at the subnational level can be visualised in Fig. 3, which plots the availability estimates at adm-1, where each dot represents a single administrative unit, considering the population size and the number of hospitals available. This figure highlights the variability in the availability of services both between and within countries. For example, many of the administrative units with larger population sizes exceed the target of 1 hospital per 500,000 population, irrespective of country. However, in some countries, there are some administrative units with very large population sizes not meeting the target (i.e. the Oromia and Amhara regions of Ethiopia) (Fig. 3). In contrast to this, there are other countries with far less populous administrative units, exceeding the target by up to 11 times.

**Fig. 4:**
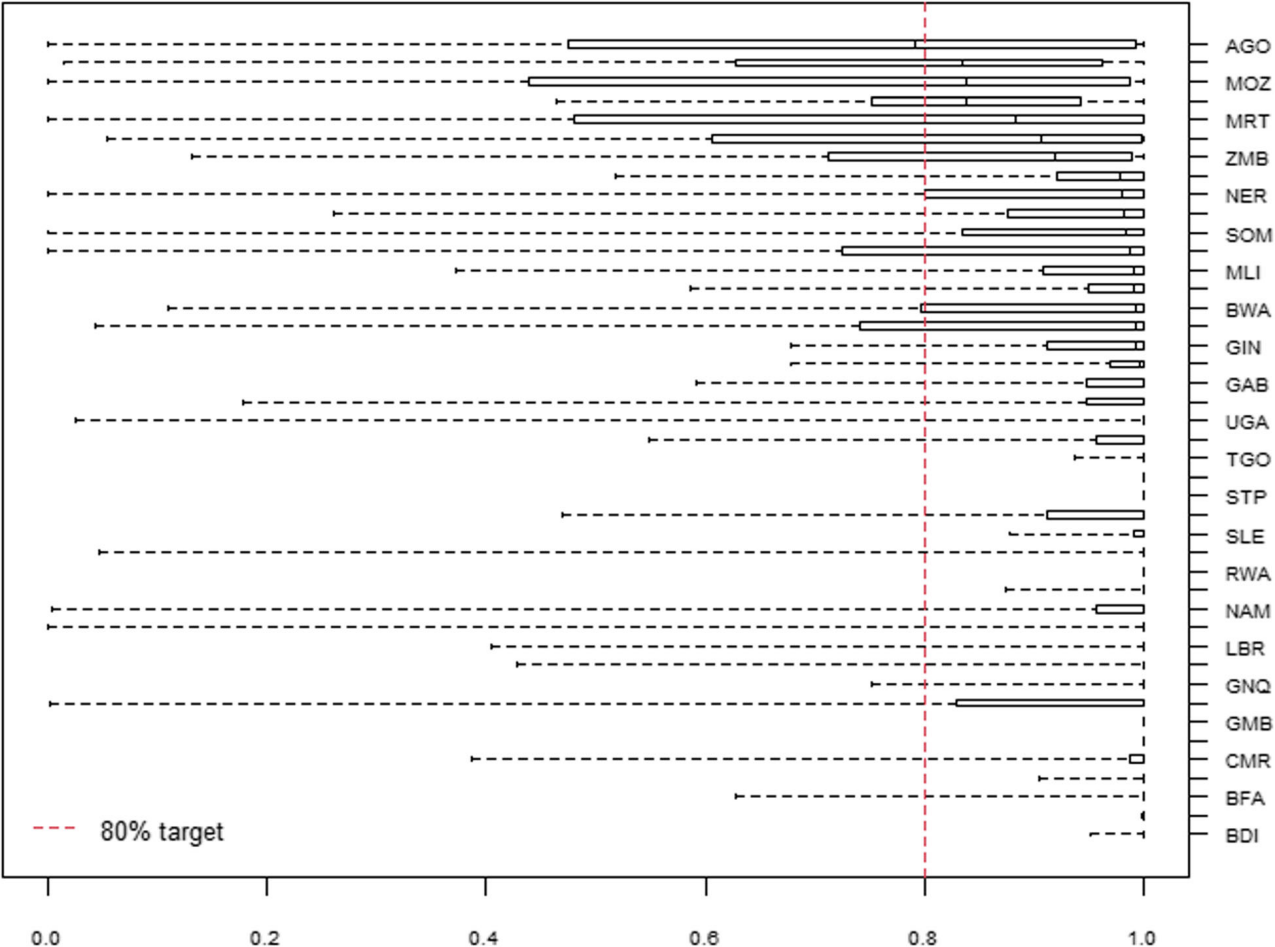
A box plot showing the variation in the median, upper and lower quartiles, and outlying data values of the proportion of pregnancies within 2-h travel time of the nearest hospital, ordered by median value (adm-2 level)

We estimated the geographical accessibility of hospitals for 45 countries for national and adm-1 levels and 43 at adm-2. At the national level, all countries were found to meet the geographical accessibility target (80% of all pregnancies located within 2-h travel time of the nearest hospital), with the exception of Eritrea (71%) (Additional file 5). Again, at sub-national administration levels, we identified substantial variability, with 56% of the countries at adm-1 and 74% at adm-2 having at least one administration unit not meeting the target. With increasing administrative units, a clear geographical pattern emerges, where areas in the north and north-east as well as central SSA are identified to have increasing areas at sub-national levels, not meeting the target (Fig. 2). National-level summary statistics can be visualised in Fig. 4, showing the median, upper, and lower quartiles, and outlying data values, of the proportion of pregnancies within 2-h of the nearest hospital at adm-2. At this level, although many of the units meet the target, large geographical variations can be observed, where, for example, in Angola, Mozambique and Mauritania, more than half of the units at adm-2 do not meet the target, and only 11 countries out of 43 have all their admin units above the 80% threshold.

To examine how the estimates of availability and geographical accessibility vary geographically, the results from the availability analysis were compared with the results from the geographical accessibility analysis. At the national level, 42 (93%) of the countries were found to meet both targets, with just one country identified as meeting the availability but not the geographical accessibility target, and two countries as meeting the geographical accessibility but not the availability target (Additional file 6). At adm-1, 12 countries (27%) were identified to have units not meeting both targets, 18 countries (40%) to have units meeting the availability but not the geographical accessibility target, and 34 countries (76%) to have units meeting the geographical accessibility but not the availability target (Additional file 7). Then at adm-2, 23 countries (53%) were identified to have units not meeting both targets, with 26 countries (60%) to have units meeting the availability but not the geographical accessibility target, and 40 countries (93%) to have units meeting the geographical accessibility but not the availability target (Additional file 8).

## Discussion

With increasingly available and reliable spatial data at finer resolutions [48], the geographical coverage of health care services can now be monitored with increasing accuracy, a key consideration towards the achievement of UHC [49]. Using recently compiled hospital location data for 45 countries across SSA [26], and high-resolution gridded population and pregnancy count estimates [50], we explore for the first time how analysis at varying spatial scales can uncover areas of need previously undetected. While considering the results, it is important to note that we use population to estimate the availability of services, as stated by the guidelines [27], and pregnancies for measuring physical access to emergency obstetric and newborn care in low-income and middle-income countries, as proposed by Ebner et al. [19]. Our results estimate that across SSA, there is an average availability of two hospitals per 500,000 people, with 93% of pregnancies occurring within 2 h of the nearest hospital, and at the national level, the results again indicate adequate coverage, with just a few countries not meeting the targets. However, when calculated at sub-national levels, in Angola and Malawi for example, it is clear that there are increasing areas of need not meeting the target, 11% and 18% at adm-1, and 32% and 82% at the adm-2, respectively, showing how the national averages usually reported for monitoring purposes can mask significant variation.

These findings highlight the importance of monitoring at sub-national levels, and while indicators exist for sub-national evaluation [8], clearer guidance on how to calculate these is needed. For example, it is not specified whether to consider a minimum population size when calculating the geographical distribution of hospitals at subnational levels, an important consideration in the assessment of health system coverage, where the monitoring of smaller administrative areas will naturally concern lower populations. Additionally, the suitability of using population as recommended in the guidelines [8], rather than women of childbearing age, births or pregnancies [17], is considered misleading [14] as it cannot accurately reflect the population at risk. This may arguably under-estimate health system coverage; however, it does not account for variations in the spatial demographics of women of childbearing age, for which the availability of emergency obstetric care is most needed. Furthermore, the guidelines do not include an explicit measure of accessibility; rather, accessibility is measured implicitly in the calculation of the availability of services at sub-national levels, and cannot truly reflect the time taken for women to them.

To further consider the accessibility of services, we measure the travel time taken to reach the nearest hospital, as recommended by global guidelines [11]. However, the definition of geographical accessibility as used in this paper [11], may likely overestimate coverage, due to the assumptions made in the estimation of travel time. For example, the geographical accessibility surface assumes fixed travel speeds (maximum speed) for specific road and land cover types [37, 51]. However, this is not a true reflection of the ground situation, where road conditions can vary considerably between and within countries, as well as according to the season, often resulting in a reduction in speed where road conditions are poor [28, 52]. The geographical accessibility surface additionally assumes that people travelling on primary, secondary, and tertiary roads have access to motorised transport that travel at the assumed speeds; however, for many, access to motorised transport is limited [37].

Furthermore, the 2-h travel time recommended by the indicator has been critiqued as a benchmark for geographical accessibility, and literature references a “golden hour” where access to emergency obstetric care within the first hour can dramatically decrease adverse outcomes and improve chances of survival for women and their children [53]. Reducing the 2-h benchmark to consider this “golden hour” would have shown a reduction in service coverage, resulting in an increase in the number of areas not meeting this target and number of women at risk. Furthermore, using a minimum threshold of at least 80% population coverage within this 2-h travel time is problematic, where in the context of the SDG “leave no one behind” agenda, it implies that it is acceptable that a fifth of population at risk do not have access to critical services. It is additionally important to note that any measure of geographical accessibility does not consider barriers related to the availability, affordability, acceptability, and appropriateness of services; factors which have shown to contribute substantially to delays in receiving care [54–56], even when geographical accessibility might otherwise be considered adequate.

We additionally assume, as in Ouma et al. [20], when measuring geographical accessibility, that hospitals provide CEmOC. This is stressed as a key limitation to the study since previous research has found that health facilities in SSA do not always provide the required number of signal functions to be classified as CEmOC [28]. As a result, it is likely that we overestimate both the availability and accessibility of services. However, as it is the most complete and up to date record of public hospitals in SSA, it provides a considerable opportunity to evaluate the availability and geographical accessibility of hospitals at a scale not achieved before, and thus, providing valuable insight into the performance of these indicators at sub-national levels. Future work should therefore evaluate the services provided by each facility, to assess whether the use of hospitals, as defined in this analysis, can accurately reflect the key signal functions outlined in the guidelines for monitoring EmOC [8]. Further analysis should additionally consider the use of more comprehensive facility datasets, including health facilities, private hospitals, and hospitals providing only specialised services [57].

## Conclusions

In exploring how the analysis of data at increasing spatial resolutions can indeed uncover areas of need overlooked, we demonstrate how the use of different input data, methods of analysis, and most pertinently, how the construction of the indicators themselves, can considerably impact monitoring outcomes. Given these caveats, the definitions of both the availability and geographical accessibility indicators should be reviewed, harmonised and standardised, to effectively measure progress towards the SDG within the context of UHC.

## Supplementary information

**Additional file 1: Table 1*.*** A descriptive table of the countries studied, their total area, total population, and total number of adm1 and adm2 units.

**Additional file 2: Table 2.** Shows the availability of hospitals at the national level (number of hospitals per 500 000 population), and the corresponding percentage of the target achieved.

**Additional file 3: Table 3.** Shows the proportion of regions not meeting the availability target at adm-1, along with their associated ASR grouping to signify comparability between countries.

**Additional file 4: Table 4.** Shows the proportion of regions not meeting the availability target at adm-2, along with their associated ASR grouping to signify comparability between countries.

**Additional file 5: Table 5.** Shows the total number of pregnancies, number of pregnancies within 2-h of the nearest hospital, and relative proportion of pregnancies within 2-h of the nearest hospital, at the national level.

**Additional file 6: FigureS1.** Compares the availability and accessibility indicator estimates at the national level.

**Additional file 7: Table 6.** Compares the number of countries and regions meeting each of the classifications at adm-1.

**Additional file 8: Table 7.** Compares the number of countries and regions meeting each of the classifications at adm-2.

## Data Availability

The datasets analysed during the current study are available from the following repositories: • Sub-Saharan Public Hospitals Geo-coded database, https://dataverse.harvard.edu/dataset.xhtml?persistentId=doi:10.7910/DVN/JTL9VY [20]. • Worldpop Pregnancy distributions, https://www.worldpop.org/project/categories?id=6 [34]. • GADM Administrative boundaries, https://gadm.org/data.html [41]. • Travel-time raster, https://figshare.com/articles/National_and_subnational_variation_in_pattern_of_febrile_case_management_in_sub-Saharan_Africa/7160363 [32].

## Abbreviations

ASR: Average spatial resolution
CEmOC: Comprehensive emergency obstetric care
EmOC: Emergency obstetric care
GADM: Global ADMinistrative areas
MMR: Maternal mortality ratio
SDG: Sustainable Development Goals
SSA: Sub-Saharan Africa
UHC: Universal health coverage
WHO: World Health Organization
WoCBA: Women of childbearing age
