# Supplementary figures and images for: Measuring the availability and geographical accessibility of maternal health services across sub-Saharan Africa
Source: BMC Med. 2020 Sep 8;18:237. doi: 10.1186/s12916-020-01707-6 (PMC7487649; doi:10.1186/s12916-020-01707-6)

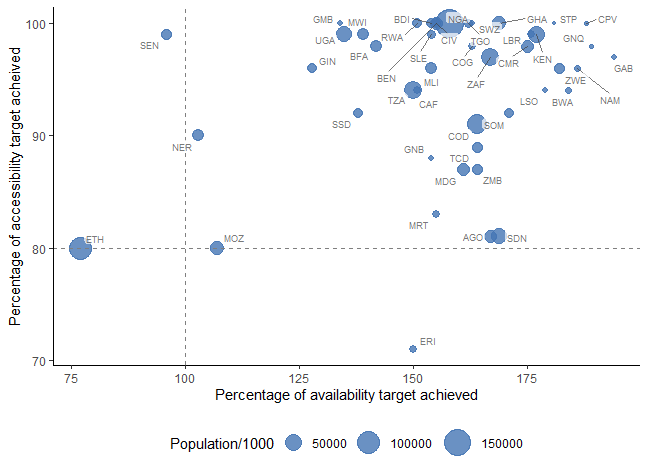

Supplement: Supplementary file 6 — Additional file 6: FigureS1. Compares the availability and accessibility indicator estimates at the national level. [file 12916_2020_1707_MOESM6_ESM.png]
